# Secondary Frequency Modulation Strategy for SiC Inverters Based on Periodic Spread Spectrum Modulation

**DOI:** 10.3390/s25041269

**Published:** 2025-02-19

**Authors:** Yanfei Cao, Junjie Su, Yan Yan, Zhichen Lin, Tingna Shi

**Affiliations:** College of Electrical Engineering, Zhejiang University, Hangzhou 310027, China; caoyanfei@zju.edu.cn (Y.C.); 22210109@zju.edu.cn (J.S.); yan_yan@zju.edu.cn (Y.Y.); linzhichen@zju.edu.cn (Z.L.)

**Keywords:** spread spectrum modulation, periodic PWM, conducted EMI, ripple, loss

## Abstract

This paper aims to design the modulation strategy of SiC motor controller for vehicles with low EMI, low device loss and low voltage/current ripple. The equivalent evaluation model of input voltage, output current and switching loss of the inverter under periodic spread spectrum modulation strategy is constructed, and the quantitative relationship between each parameter of spread spectrum modulation and the three indicators is established. The input/output performance and loss level of the inverter under different spread spectrum modulation strategies are evaluated. On this basis, based on the carrier frequency distribution characteristics of periodic signal spread spectrum modulation, a “secondary frequency modulation” strategy is proposed to reduce the inverter-conducted EMI to a greater extent under the limited spread spectrum range. Experimental results show that compared with the single periodic signal spread spectrum modulation, the “secondary frequency modulation” strategy can reduce the peak value of inverter-conducted EMI to a greater extent without increasing the ripple and loss of the inverter.

## 1. Introduction

As a third-generation semiconductor material, SiC has excellent characteristics such as high temperature resistance, high voltage resistance, high switching frequency and low loss, which makes it useful in electric vehicle motor controllers. However, the high-frequency switching operation of SiC MOSFETs is prone to deteriorate the electromagnetic compatibility characteristics of the controller, and it is of great significance to make full use of the advantages brought about by the high-frequency characteristics of SiC, while suppressing its negative impacts to enhance the performance of electric vehicle motor controllers.

Because of the characteristic of “dispersing the energy spikes”, spread spectrum modulation strategy is widely used to reduce the conducted electromagnetic interference (EMI) in motor controllers. The principle is to change the carrier wave, so that the energy of the concentrated part of the system can be dispersed to a wider frequency range. According to Parseval’s theorem, if the energy distribution of a signal in the time domain is unchanged, the energy of the signal in the frequency domain is also unchanged. Therefore, under the precondition that the signal energy in the frequency domain remains unchanged, spread spectrum modulation realizes the beneficial effect of reducing signal spikes in a wider frequency range by expanding the bandwidth of the signal energy distribution [1]. Common spread spectrum modulation strategies can be divided into two main categories: periodic (PWM) [1,2,3,4] and random (PWM) [4,5,6,7], which are realized by injecting periodic or random signals into the carrier, respectively. Common periodic signals include: sinusoidal signals, triangular signals, sawtooth signals, etc. [6,8]. Random signals are usually replaced by chaotic signals, and common chaotic sequences include: logistic sequences, cubic sequences, and so on. Of these two types of methods, the principle of periodic PWM is relatively simple, has less impact on the dynamic performance of the motor system, and is easily realized in engineering, which makes it an important solution to improve the inverter conduction EMI problem [9]. Existing research on EMI reduction of spread spectrum modulation mainly focuses on the influence of different modulation parameters on the spread spectrum effect, for example, the effects of different random signal types, periodic signal types, frequency and spreading width of periodic signals and other parameters on the spreading effect [8,10,11].

However, in addition to conducted EMI, the design of modulation strategies and the performance evaluation of inverters usually consider the following aspects: the input/output performance of the inverter [12,13], the volume and weight of the inverter [12,14,15,16], and the overall efficiency of the inverter [14,15,16]. The input/output performance of the inverter is mainly determined by the modulation technology and the topology structure of the inverter [13,15,16]. The performance can be improved by adding auxiliary devices to the topology structure or optimizing the modulation technology. For example, the coupled reactor and hybrid modulation technology are adopted in literature [12], and impedance network is adopted in literature [13], to achieve better modulation effects. When the topology structure and modulation technology are determined, the volume, weight and overall efficiency of the inverter are usually affected by the power device itself [14,16]. For example, reference [14] includes experimental studies on two kinds of inverters with full SiC and Si power devices. The results show that the application of SiC devices can help to provide higher efficiency and smaller heat sink volume. Reference [16] reports experimental studies on two kinds of high-power three-phase two-level voltage source inverters of SiC and Si respectively, and finally shows that the weight of the inverter component designed based on SiC was reduced by about 39%.

Therefore, specifically, the indicators that need to be considered for the modulation strategy also include input-side DC voltage ripple [17], output current ripple [18,19], and switching losses. The input voltage ripple affects the output voltage waveform quality and the design of the DC side support capacitor, and excessive voltage ripple has higher requirements for the design of the support capacitor [17], which is not conducive to the miniaturization and lightweighting of the inverter. The output current ripple directly affects the efficiency of the loaded motor, and it will also lead to the reduction of the control accuracy of the motor controller. The magnitude of switching loss directly affects the overall efficiency of the inverter. For these indicators, there is a lack of a comprehensive analysis of the impact brought by spread spectrum modulation, which is not conducive to further improving the performance of motor controllers through the optimization of modulation strategies.

This paper aims to design a modulation strategy for electric vehicle motor controllers with low EMI, low device loss, and low voltage/current ripple. Based on the waveform characteristics of the periodic signals, this paper intends to analyze the spectral distribution characteristics of the conducted EMI; and based on the calculation of voltage ripple and flux fluctuation amount, this paper establishes an equivalent analysis model for the input voltage ripple and the output current ripple. This paper further evaluates the level of inverter loss by analyzing the number of carrier waves per unit of time for different spread spectrum modulation strategies, so that a comprehensive analysis of the inverter input and output performance under the spread spectrum modulation strategy is realized. Based on the conclusions of the analysis, a new modulation strategy of “secondary frequency modulation” is proposed, and a comparative experimental study is carried out between the proposed strategy and the existing spread-spectrum modulation strategies in terms of voltage/current ripple, loss, and conducted EMI spikes.

## 2. Traditional Spread Spectrum Modulation Strategy

### 2.1. Inverter Topology

**Figure 1:**
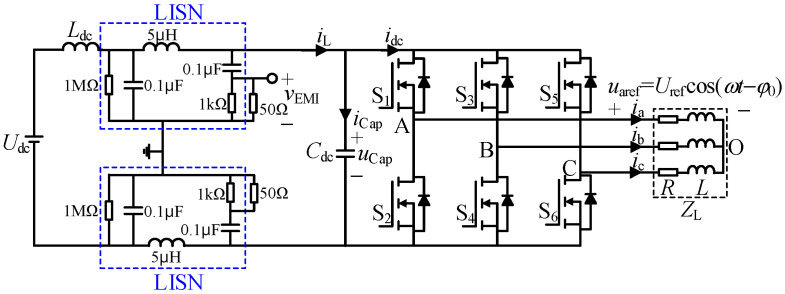
Topology of two-level three-phase system with LISN.

Figure 1 shows the three-phase two-level inverter topology adopted by electric vehicle motor controllers, which also includes the Line Impedance Stabilization Network (LISN), which is used to test the conducted EMI of switching circuits according to CISPR 25 standard. The three-phase reference voltage of the inverter is defined as:

(1) $$\left\{\begin{array}{l} u_{\mathrm{aref}}(t)=U_{\mathrm{ref}}\cos\left(\omega t-\varphi_{0}\right)\\ u_{\mathrm{bref}}(t)=U_{\mathrm{ref}}\cos\left(\omega t-2\pi /3-\varphi_{0}\right)\\ u_{\mathrm{cref}}(t)=U_{\mathrm{ref}}\cos\left(\omega t+2\pi /3-\varphi_{0}\right) \end{array}\right.$$

where *U*_ref_ = *m*·*U*_dc_/1.732 is the reference phase voltage amplitude; *m* is the modulation index; ω = 2π*f*_1_ is the reference angular frequency; *f*_1_ is the fundamental frequency; *φ*_0_ is the initial phase. Set *s*_a_, *s*_b_ and *s*_c_ as switching functions, and their values are 1 when the corresponding upper bridge arm is opened, and 0 when it is closed. The inverter output phase voltage can be expressed as:

(2) $$\left[\begin{array}{l} u_{a}(t)\\ u_{b}(t)\\ u_{c}(t) \end{array}\right]=\frac{1}{3}\left[\begin{array}{ccc} 2 & -1 & -1\\ -1 & 2 & -1\\ -1 & -1 & 2 \end{array}\right]\left[\begin{array}{l} s_{a}(t)\\ s_{b}(t)\\ s_{c}(t) \end{array}\right]U_{\mathrm{dc}}$$

Since the output side of the inverter is connected to inductive load, the output current waveforms are in the form of sine, and their expressions are:

(3) $$\left\{\begin{array}{l} i_{a}(t)=I_{o}\cos(\omega t-\varphi_{0}-\varphi_{L})\\ i_{b}(t)=I_{o}\cos\left(\omega t-2\pi /3-\varphi_{0}-\varphi_{L}\right)\\ i_{c}(t)=I_{o}\cos\left(\omega t+2\pi /3-\varphi_{0}-\varphi_{L}\right) \end{array}\right.$$

where *I*_o_ is the amplitude of output phase current; *φ*_L_ is the load power factor angle; the expression for the DC input current *i*_dc_ is:

(4) $$i_{\mathrm{dc}}=s_{a}\cdot i_{a}+s_{b}\cdot i_{b}+s_{c}\cdot i_{c}$$

### 2.2. Implementation Process of Traditional Spread Spectrum Modulation Strategy

**Figure 2:**
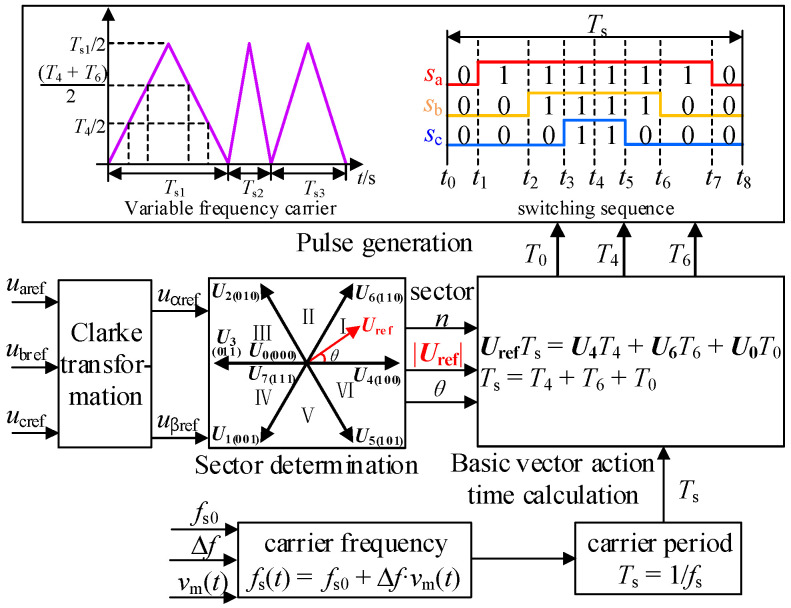
Implementation process of traditional periodic spread spectrum modulation.

The implementation process of traditional spread spectrum modulation is shown in Figure 2. Compared with the fixed-carrier-frequency SVPWM modulation, the two methods are the same in the calculation of the action time of each vector, but there are differences in the carrier.

Spread spectrum modulation adopts carriers with varying frequencies, and its frequency expression can be uniformly written as:

(5) $$f_{s}(t)=f_{s0}+\Delta f\cdot v_{m}(t)$$

where *f*_s_(*t*) is the carrier frequency; *f*_s0_ is the carrier center frequency; Δ*f* is the maximum carrier frequency deviation. *v*_m_(*t*) is a periodic signal, and the expressions of sine, triangle and sawtooth three periodic signals are:

(6) $$v_{m\text{\_}\sin}(t)=\sin(2\pi f_{m}t)\;,\;0\leq t\leq T_{m}$$

(7) $$v_{m\text{\_}\mathrm{tri}}(t)=\left\{\begin{array}{l} 4f_{m}t-1,\;0\leq t<T_{m}/2\\ -4f_{m}t+3\;,T_{m}/2\leq t\leq T_{m} \end{array}\right.$$

(8) $$v_{m\text{\_}\mathrm{saw}}(t)=2f_{m}t-1\;,\;0\leq t\leq T_{m}$$

where *f*_m_ is the frequency of the periodic signal and *T*_m_ = 1/*f*_m_ is the period value of the periodically modulated signal.

## 3. Performance Analysis of Spread Spectrum Modulation Strategy

In order to achieve the modulation optimization under multi-objective, this section analyzes the effects of periodic signal waveform characteristics on EMI, inverter switching loss, and voltage/current ripple. Based on the analysis method of each indicator under fixed carrier frequency modulation, the relevant conclusions of ripple and loss under spread spectrum modulation strategy are obtained.

### 3.1. Conducted EMI Analysis of the Inverter Under Spread Spectrum Modulation

**Figure 3:**
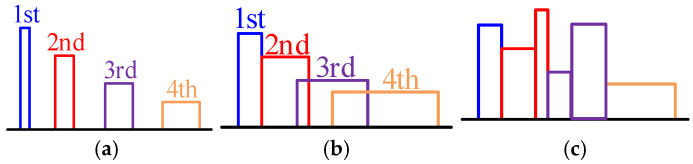
Diffusion of each frequency band for different Δ*f*: (**a**) Spread spectrum in a small area; (**b**) Spread spectrum over a wide area; (**c**) Spectrum overlap.

The factors that affect the peak of the conducted EMI spectrum are mainly the characteristics of the carrier frequency. It is manifested in the following two points:

① When spread spectrum modulation is adopted, the peak of EMI spectrum at *hf*_s0_ will be mainly dispersed in the range of [*h*(*f*_s0_ − Δ*f*), *h*(*f*_s0_ + Δ*f*)] [5], *h* = 1, 2, 3…. As shown in Figure 3, the main factor affecting the diffusion degree of the EMI spectrum is Δ*f*; the larger Δ*f* is, the wider the diffusion degree of EMI spectrum is, and the more it can weaken the EMI spectrum peak to a greater extent. However, only increasing Δ*f* to reduce the EMI peak may lead to the phenomenon of “spectrum overlap” [20]; that is, the originally disjoint frequency bands overlap after the spread spectrum, making the EMI energy of some frequency bands rise, as shown in Figure 3b,c.

**Figure 4:**
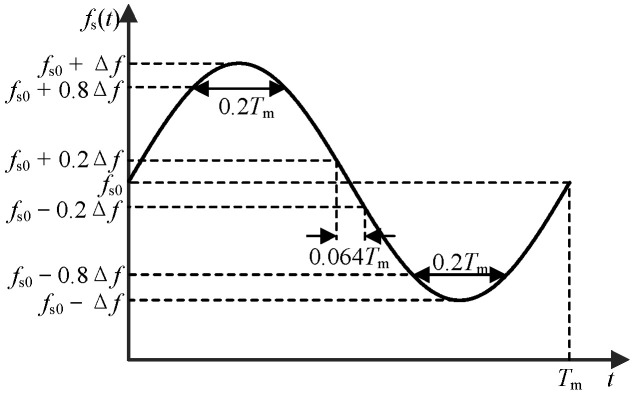
The duration of different frequency bands when *v*_m_(*t*) is sinusoidal signal.

② For periodic signal *v*_m_(*t*), the distribution of carrier frequency in different frequency bands is affected by the gradient of periodic signal [21], which affects the spectrum distribution of EMI. Take the sinusoidal signal as an example: as shown in Figure 4, the absolute value of the gradient of the sinusoidal signal at the value of 0 is the largest, the carrier frequency value is *f*_s0_, and the action time of *f*_s0_ and its nearby frequency bands is the shortest. The gradient of the sinusoidal signal at the extreme value is 0, and the carrier frequency value is *f*_s0_ ± Δ*f*; *f*_s0_ ± Δ*f* and its nearby frequency band have the longest action time. Therefore, when *v*_m_(*t*) is the sinusoidal signal, the frequency distribution of the carrier will present a trend of “high on both sides and low in the middle”.

**Figure 5:**
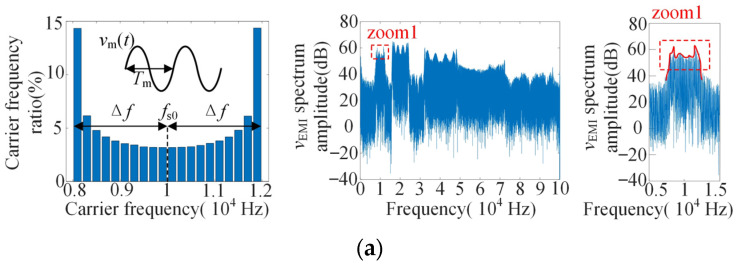

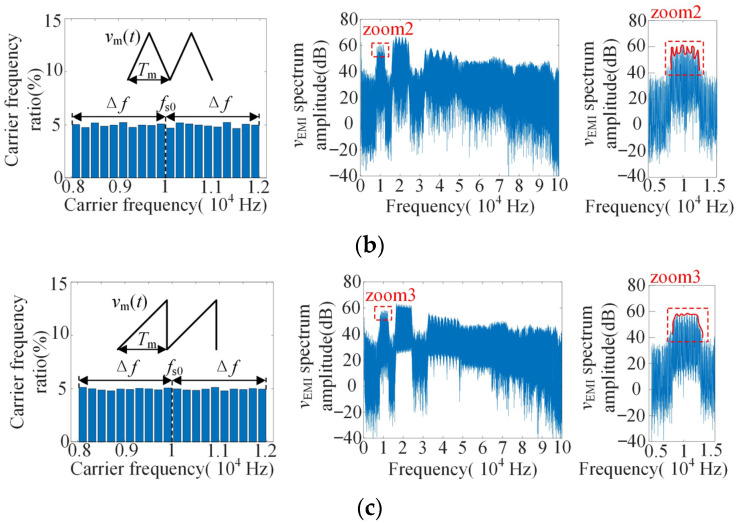
Carrier frequency distribution and DFT spectrum of *v*_EMI_ under different *v*_m_(*t*) waveforms: (**a**) *v*_m_(*t*)-sine; (**b**) *v*_m_(*t*)-triangle; (**c**) *v*_m_(*t*)-sawtooth.

The voltage on the output impedance of LISN (i.e., *v*_EMI_ in Figure 1) is analyzed by DFT, and its voltage spectrum is used to replace the EMI spectrum to analyze the influence of carrier frequency characteristics on EMI. At Δ*f* = 2 kHz, *m* = 0.9, *f*_s0_ = 10 kHz, *f*_1_ = 50 Hz, *f*_m_ = 30 Hz. When *v*_m_(*t*) are sine, triangle and sawtooth, the simulation results are shown in Figure 5. The figure shows the carrier frequency ratio and the corresponding EMI spectrum distribution.

As shown in the Figure 5, the distribution characteristics of *v*_EMI_ spectrum at the frequency band [*f*_s0_ − Δ*f*, *f*_s0_ + Δ*f*] is similar to that of the carrier frequency ratio.

### 3.2. Equivalent Analysis of Voltage and Current Ripple Under Spread Spectrum Modulation

#### 3.2.1. DC Side Voltage Ripple

According to Equation (2), the fluctuation of DC voltage at the input side will, on the one hand, affect the stability of output voltage. On the other hand, the higher voltage ripple has higher requirements for the design of DC support capacitor *C*_dc_ [17], which will increase the volume and weight of the controller. Therefore, it is necessary to establish a quantitative model for the influence of the spread spectrum modulation strategy on input voltage ripple.

According to the current relationship in the inverter topology in Figure 1, the capacitor current *i*_Cap_, the inductor current *i*_L_ and the DC input side current *i*_dc_ satisfy *i*_Cap_ = *i*_L_ − *i*_dc_. The current is decomposed into the sum form of the average value and the fluctuation value:

(9) $$i_{\mathrm{Cap}}=I_{\mathrm{Cap}}+i_{\mathrm{Cap}\text{\_}\mathrm{rip}},\;i_{L}=I_{L}+i_{L\text{\_}\mathrm{rip}},\;i_{\mathrm{dc}}=I_{\mathrm{dc}}+i_{\mathrm{dc}\text{\_}\mathrm{rip}}$$

where *I*_Cap_, *I*_L_ and *I*_dc_ represent the average current value of capacitor *C*_dc_, the stray inductor *L*_dc_, and *i*_dc,_ respectively. *i*_Cap_rip_, *i*_L_rip_ and *i*_dc_rip_ represent the corresponding current ripple components. Among them, the relationship of current ripple components satisfies *i*_Cap_rip_ = *i*_L_rip_ − *i*_dc_rip_. When the DC filter capacitor is large enough, almost all the ripple components of the input current of the inverter flow through the capacitor, and the ripple current *i*_L_rip_ is negligible compared with the ripple current *i*_dc_rip_ [17], obtaining:

(10) $$i_{\mathrm{Cap}\text{\_}\mathrm{rip}}=i_{L\text{\_}\mathrm{rip}}-i_{\mathrm{dc}\text{\_}\mathrm{rip}}\approx -i_{\mathrm{dc}\text{\_}\mathrm{rip}}=I_{\mathrm{dc}}-i_{\mathrm{dc}}$$

(11) $$u_{\mathrm{Cap}\text{\_}r}=\frac{1}{C_{\mathrm{dc}}}\int_{0}^{T_{s}}i_{\mathrm{Cap}\text{\_}\mathrm{rip}}dt=\frac{1}{C_{\mathrm{dc}}}\int_{0}^{T_{s}}\left(I_{\mathrm{dc}}-i_{\mathrm{dc}}\right)dt$$

where *u*_Cap_r_ is the input voltage ripple.

It can be seen from Equation (11) that the ripple is the integral of the fluctuation quantity with respect to time. Under the spread spectrum modulation, the change of carrier period will affect the input and output waveform ripple.

According to Equations (3) and (4), *i*_dc_ is determined by *i*_a_, *i*_b_ and *i*_c_, and is a cosine function with amplitude *I*_o_, denoted *i*_dc_ as *I*_o_·cos[*α*(*t*)], and *α*(*t*) is the phase of the cosine function with time, which is determined by *i*_a_, *i*_b_ and *i*_c_. Ideally, *I*_o_ satisfies $I_{o}=U_{\mathrm{ref}}/\sqrt{R^{2}+{\left(2\pi f_{1}L\right)}^{2}}$, where *R* and *L* are the resistance and inductance in the load impedance *Z*_L_, respectively; therefore, Equation (11) can be expanded as:

(12) $$u_{\mathrm{Cap}\text{\_}r}=\frac{1}{C_{\mathrm{dc}}}\int_{0}^{T_{s}}\left\{I_{\mathrm{dc}}-I_{o}\cos\left[\alpha (t)\right]\right\}dt=\frac{1}{C_{\mathrm{dc}}}\int_{0}^{T_{s}}\left\{I_{\mathrm{dc}}-\frac{m\cdot U_{\mathrm{dc}}/\sqrt{3}}{\sqrt{R^{2}+{\left(2\pi f_{1}L\right)}^{2}}}\cos\left[\alpha (t)\right]\right\}dt$$

The average value of *i*_dc_ is expressed as:

(13) $$I_{\mathrm{dc}}=\frac{1}{T_{s}}\int_{0}^{T_{s}}i_{\mathrm{dc}}(t)dt$$

So that the voltage ripple expression can be obtained.

According to Equation (11), the DC voltage ripple at the input side can be calculated theoretically, and the quantitative relationship between voltage ripple and each modulation parameter can be established. According to Equation (12), the factors affecting voltage ripple are the integration time *T*_s_ and the amplitude of the integration current. Here, *T*_s_ = 1/*f*_s_(*t*) = 1/[*f*_s0_ + Δ*f*·*v*_m_(*t*)], and the amplitude of the integrated current is related to *m* and *f*_1_. Therefore, according to the above derivation process, the modulation parameters affecting voltage ripple are *f*_s0_, Δ*f*, *v*_m_(*t*) (including *f*_m_), *m* and *f*_1_, respectively.

#### 3.2.2. AC Side Output Current Ripple

**Figure 6:**
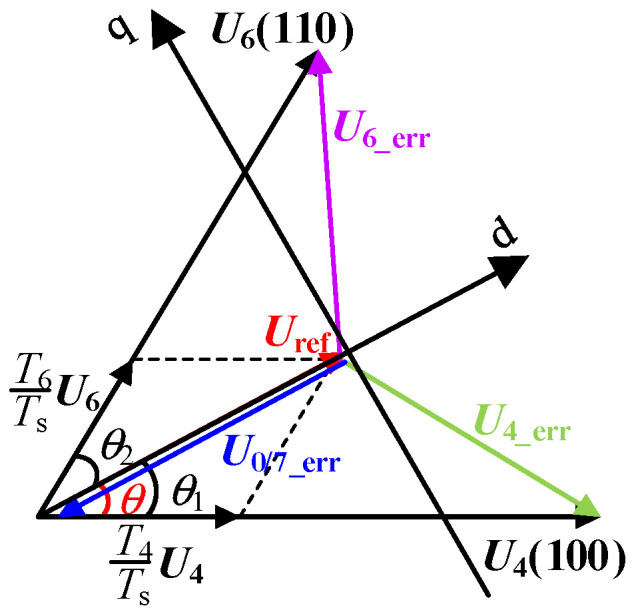
Schematic diagram of voltage error vector in sector I.

In this section, in order to achieve fast and accurate evaluation of inverter output waveform quality under different modulation methods, the effective value model of motor stator flux fluctuation under the spread spectrum modulation strategy is constructed, which is equivalent to the sum of three-phase output current fluctuation, and can be used to analyze and compare the influence of different periodic signals and spread spectrum modulation parameters on inverter output waveform quality. The steps are as follows: in the vector synthesis process, there is an error voltage vector between the basic vector and the reference vector at any time [18,19], and the reference voltage vector is defined as ***U*_ref_**. Without loss of generality, when the reference vector ***U*_ref_** is located in sector I, the corresponding error voltage vectors are ***U*_4_err_** and ***U*_6_err_**. When the basic voltage vectors ***U*_4_** and ***U*_6_** act is shown in Figure 6.

The expressions of the error voltage vectors ***U*_4_err_**, ***U*_6_err_** and ***U*_0_err_** are:

(14) $$\left\{\begin{array}{l} U_{0\text{\_}\mathrm{err}}=U_{0}-U_{\mathrm{ref}}\\ U_{4\text{\_}\mathrm{err}}=U_{4}-U_{\mathrm{ref}}\\ U_{6\text{\_}\mathrm{err}}=U_{6}-U_{\mathrm{ref}} \end{array}\right.$$

The direction of the reference vector ***U*_ref_** is defined as the d-axis, and the direction of the leading d-axis π/2 is defined as the q-axis. The error voltage vectors ***U*_4_err_**, ***U*_6_err_** and ***U*_0_err_** are decomposed into the d-axis and the q-axis:

(15) $$\left\{\begin{array}{l} U_{6\_\mathrm{err}}=(\frac{2}{3}U_{\mathrm{dc}}\cos\theta_{1}-U_{\mathrm{ref}})-j\frac{2}{3}U_{\mathrm{dc}}\sin\theta_{1}\\ U_{4\text{\_}\mathrm{err}}=(\frac{2}{3}U_{\mathrm{dc}}\cos\theta_{2}-U_{\mathrm{ref}})+j\frac{2}{3}U_{\mathrm{dc}}\sin\theta_{2}\\ U_{0\text{\_}\mathrm{err}}=-U_{\mathrm{ref}} \end{array}\right.$$

Define *u*_d0_~*u*_d2_ and *u*_q1_~*u*_q2_ as:

(16) $$\left\{\begin{array}{l} u_{d0}=-U_{\mathrm{ref}}\\ u_{d1}=\frac{2}{3}U_{\mathrm{dc}}\cos\theta_{1}-U_{\mathrm{ref}},\;u_{q1}=-\frac{2}{3}U_{\mathrm{dc}}\sin\theta_{1}\\ u_{d2}=\frac{2}{3}U_{\mathrm{dc}}\cos\theta_{2}-U_{\mathrm{ref}},\;u_{q2}=\frac{2}{3}U_{\mathrm{dc}}\sin\theta_{2} \end{array}\right.$$

According to the motor voltage equation, the integral of the error voltage vector with respect to time within the carrier period *T*_s_ is the stator flux fluctuation *ψ*_rip_, as shown in the following equation:

(17) $$\Psi_{\mathrm{rd}}=\int_{0}^{T_{s}}u_{d}\;dt,\Psi_{\mathrm{rq}}=\int_{0}^{T_{s}}u_{q}\;dt$$

Among them, *u*_d_ and *u*_q_ are error voltage components of d-axis and q-axis in different time periods, and their values in different time periods can be represented by *u*_d0_~*u*_d2_ and *u*_q1_~*u*_q2_ respectively, as shown in Equation (16).

Furthermore, the effective value *ψ*_rip_rms_ of the flux fluctuation quantity in time *T* can be calculated as:

(18) $$\Psi_{\mathrm{rip}\text{\_}\mathrm{rms}}=\sqrt{\frac{1}{T}\int_{0}^{T}\left(\Psi_{\mathrm{rd}}^{2}+\Psi_{\mathrm{rq}}^{2}\right)\;dt}$$

Among them, to ensure that *f*_s_(*t*) and *i*(*t*) contained in the selected time period are all integer periods, *T* should take at minimum the least common multiple of 1/*f*_m_ and 1/*f*_1_.

According to Equations (16) and (17), the amplitude of flux fluctuation is affected by modulation parameters similar to input voltage ripple, which is mainly determined by the integration time *T*_s_ (*T*_s_ = 1/[*f*_s0_ + Δ*f*·*v*_m_(*t*)]) and the amplitude of the integral component. According to Equation (16), the integral components *u*_d_ and *u*_q_ are mainly determined by the amplitude |***U*_ref_**|, since the modulation parameters affecting *ψ*_rip_rms_ are *f*_s0_, Δ*f*, *v*_m_(*t*) (including *f*_m_) and *m*, respectively.

#### 3.2.3. Analysis of the Influence of Spread Spectrum Modulation Parameters on Ripple

In this section, under the conditions of *U*_dc_ = 600 V, *R* = 2.75 Ω, *L* = 1 mH, *C*_dc_ = 1680 μF, the input voltage ripple and flux fluctuation are calculated according to the derived expressions.

The initial values of the five modulation parameters affecting the two indicators are set as follows: *f*_s0_ = 10 kHz, Δ*f* = 0 kHz, *f*_1_ = 50 Hz, *m* = 0.9 and *f*_m_ = 30 Hz, and any two of the five parameters are changed to present the calculation results in the form of a 3D surface graph.

**Figure 7:**
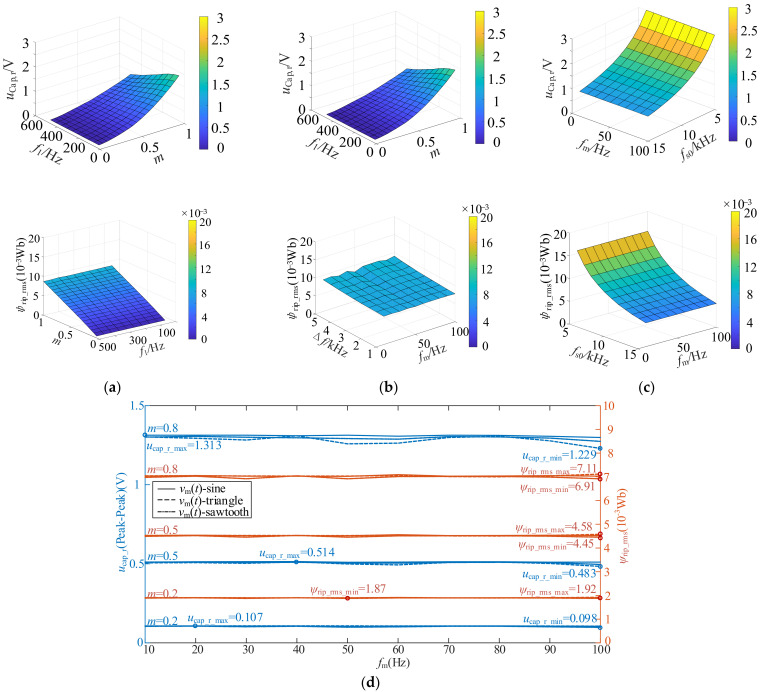
Variation trend of voltage ripple and stator flux fluctuation with different parameters: (**a**) *m* = 0.05:0.05:1, *f*_1_ = 50:50:500 Hz; (**b**) Δ*f* = 1 k:0.5 k:5 kHz, *f*_m_ = 10:10:100 Hz; (**c**) *f*_s0_ = 5 k:1 k:15 kHz, *f*_m_ = 10:10:100 Hz; (**d**) Voltage ripple and flux fluctuation values corresponding to different *v*_m_(*t*) and *f*_m_ conditions under three modulation index.

When *v*_m_(*t*) is the sawtooth signal, the calculation results are shown in Figure 7. The results are highly similar when *v*_m_(*t*) is sinusoidal signal or triangular signal. Among them, *m* = 0.05:0.05:1 means that the modulation index *m* starts from 0.05 and changes in the amplitude of 0.05 each time until *m* = 1, and the other parameters are expressed in the same way. The integration time *T* for calculating the effective value of flux fluctuation quantity in Equation (18) is 1 s. According to the results shown in Figure 7a–c, the voltage ripple and flux fluctuation are mainly affected by the four parameters of *m*, *f*_1_, Δ*f* and *f*_s0_, and are not significantly affected by *f*_m_. According to the above results, the variation rules of input voltage ripple and flux fluctuation with each parameter are shown in Table 1.

In practical applications, the carrier center frequency *f*_s0_ is usually fixed, and the modulation index *m* and fundamental frequency *f*_1_ usually change with the change of load conditions. Therefore, the only degrees of freedom that can be changed are Δ*f* and the type of periodic signal *v*_m_(*t*) (including *f*_m_).

The influence of the types of periodic signals *v*_m_(*t*) and *f*_m_ on the input voltage ripple and flux fluctuation is further quantitatively analyzed according to the above derived formula, under the three modulation indexes of *m* of 0.2 (low modulation index), 0.5 (medium modulation index) and 0.8 (high modulation index), the voltage ripple and flux fluctuation of three periodic signals of sine, triangle and sawtooth under 10 sets of parameters of *f*_m_ = 10:10:100 Hz; that is, 30 sets of calculation results correspond to each modulation condition. Two sets of results and corresponding conditions for the minimum and maximum values of input voltage ripple and flux fluctuation were compared from the 30 sets of results. The other calculated conditions were *f*_s0_ = 10 kHz, Δ*f* = 1 kHz and *f*_1_ = 50 Hz, and the specific results are shown in Figure 7d.

Therefore, by comparing the results in Figure 7, it can be seen that when the four parameters *m*, *f*_s0_, Δ*f* and *f*_1_ are all determined, *v*_m_(*t*) and *f*_m_ have little influence on the input voltage ripple and flux fluctuation. According to Figure 7d, when the waveform characteristics of *v*_m_(*t*) change, the voltage ripple is basically around 1 V, which is very limited compared to the vehicle voltage platform of 800 V. For the flux ripple, which is basically equivalent to the current ripple, its order of magnitude is 10^−3^ Wb, which is 1~2 orders of magnitude different from the permanent magnet flux amplitude of the vehicle motor. Therefore, it can be analyzed that in the vehicle scenario, the waveform characteristics of the periodic signal have little influence on the DC side voltage ripple of the inverter, and the influence on the AC side current ripple is relatively limited. When optimizing the modulation strategy, the influence of *v*_m_(*t*) waveform characteristics on ripple can be temporarily ignored or less considered.

### 3.3. Loss Analysis Under Spread Spectrum Modulation

The loss of power switching devices includes switching loss and conduction loss, and switching loss is the main one. Switching loss can be fitted as a quadratic function related to current [22]:

(19) $$E_{\mathrm{MOSFET},\mathrm{sw}}[i(t)]=A_{1}i^{2}(t)+B_{1}i(t)+C_{1}$$

where, *i*(*t*) is the current flowing through the switch tube at time *t*, and the parameters *A*_1_, *B*_1_ and *C*_1_ can be obtained by curve fitting with double-pulse experimental data. The total loss is usually determined by the single turn-on and turn-off loss of the switch tube and the total switching times per unit time [22]. In a carrier cycle, the upper and lower switches of the same bridge arm are turned on and off once, so the switching loss can be evaluated equivalently by analyzing the number of carriers included in the corresponding modulation strategy per unit time. The number of carriers included in different modulation strategies within 1 s is shown in Table 2.

As can be seen from Table 2, under different Δ*f* for different periodic signals, since *f*_s_ always fluctuates periodically around *f*_s0_, the number of carriers included in unit time is almost the same, and compared with the fixed carrier frequency condition, the number of carriers is not significantly increased, so it can be considered that the spread spectrum modulation strategy has almost no effect on the switching loss of the inverter. Therefore, based on the above analysis, the influence of modulation strategy on switching loss can be less considered when designing periodic spread spectrum modulation strategy.

### 3.4. Summary

In summary, for the three parameters of periodic signals, *v*_m_(*t*), *f*_m_ and Δ*f*, that need to be considered in the design of spread spectrum modulation strategy, it can be concluded that *v*_m_(*t*) and *f*_m_ have no significant influence on ripple and loss through the mathematical model. Under the premise of weighing multiple indicators, when designing a new spread spectrum modulation strategy, it only needs to focus on limiting Δ*f* to ensure that under the same conditions, compared with the traditional spread spectrum modulation strategy, the conducted EMI is reduced to a greater extent.

## 4. “Secondary Frequency Modulation” Spread Spectrum Modulation Strategy

**Figure 8:**
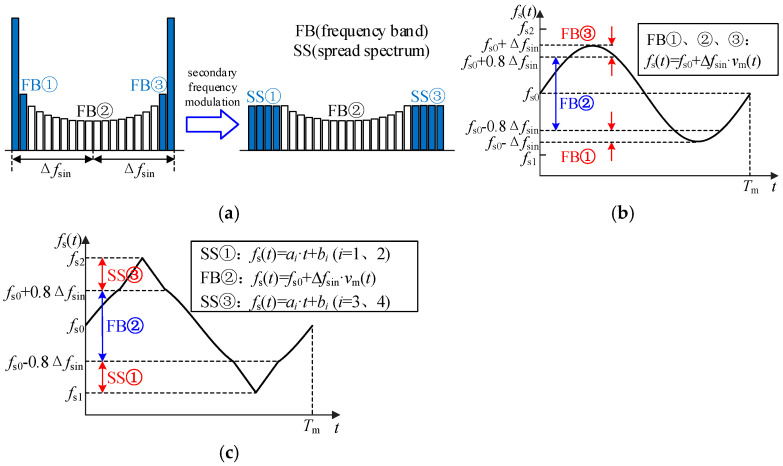
“Secondary FM” principle and implementation process: (**a**) ”Secondary FM” principle; (**b**) ”Primary frequency modulation” carrier frequency variation law and expression; (**c**) ”Secondary FM” carrier frequency variation law and expression.

According to the above analysis, this paper further proposes a “secondary frequency modulation (secondary FM)” spread spectrum modulation strategy based on sinusoidal signal waveform characteristics, which can reduce the peak value of conducted EMI to a greater extent than single periodic signal spread spectrum modulation within the same frequency fluctuation range, and does not increase additional ripple and loss. The schematic diagram of the spectrum dispersion effect is shown in Figure 8a. The main idea is explained as follows: on the basis of the spread spectrum modulation of *v*_m_(*t*) for sinusoidal signal, the concentrated part of the uneven frequency band of high-frequency and low-frequency is dispersed, so as to further reduce the conducted EMI peak. The spread spectrum modulation of a single sinusoidal signal is denoted as “primary frequency modulation”, and the modulation strategy of dispersing a specific frequency band based on the “primary frequency modulation” is denoted as “secondary FM”. The schematic diagram of carrier frequency change after secondary FM is shown in Figure 8c, and the design basis of the parameters in the figure is explained as follows:

### 4.1. Determine the Frequency Band and Diffusion Mode for Secondary Diffusion Based on the Waveform Characteristics of Periodic Signals

According to Figure 5a, when *v*_m_(*t*) is a sinusoidal signal, part of the frequency bands with uneven carrier frequency distribution are mainly concentrated on both sides of the full frequency band, and the proportion content of the other frequency bands is significantly lower than that of the triangular signal and sawtooth signal. The frequency bands are defined as follows: [*f*_s0_ − Δ*f*, *f*_s0_ − 0.8Δ*f*] for frequency band ①(FB①), [*f*_s0_ − 0.8Δ*f*, *f*_s0_ + 0.8Δ*f*] for frequency band ②(FB②), and [*f*_s0_ + 0.8Δ*f*, *f*_s0_ + Δ*f*] for frequency band ③(FB③). Through the simulation analysis, the EMI peaks of sinusoidal, triangular and sawtooth signals in different frequency bands and adjacent bands are shown in the following table. The parameters of the simulation are: *f*_s0_ = 10 kHz, *m* = 0.9, *f*_m_ = 30, *f*_1_ = 50 Hz, Δ*f* =3 kHz.

As can be seen from Table 3, when *v*_m_(*t*) is a sinusoidal signal, the peak of the *v*_EMI_ spectrum is mainly distributed in narrow FB① and FB③, and the EMI spectrum peak in FB② is significantly lower than when *v*_m_(*t*) is a triangular signal or a sawtooth signal. Based on this feature, the FB① and FB③ are further spread to further reduce the conducted EMI peak.

In order to distinguish it from the Δ*f* of the spread spectrum modulation of a single periodic signal, the frequency deviation variable of the “primary frequency modulation” of the sinusoidal signal in the “secondary FM” strategy is denoted as Δ*f*_sin_. The frequency component in the original FB① is diffused to the frequency band [*f*_s1_, *f*_s0_ − 0.8Δ*f*_sin_], and [*f*_s1_, *f*_s0_ − 0.8Δ*f*_sin_] is denoted as spread spectrum ①(SS①). FB② remains unchanged. The FB③ is spread to [*f*_s0_ + 0.8Δ*f*_sin_, *f*_s2_] band, [*f*_s0_ + 0.8Δ*f*_sin_, *f*_s2_] is denoted as spread spectrum ③(SS③). *f*_s1_ and *f*_s2_ are the lower and upper limits of the fundamental carrier frequency band of “secondary FM”, respectively. According to Figure 8c, after “secondary FM”, the variation law of the carrier in the two parts of the SS① and SS③ is a piecewise primary function, and *a_i_* and *b_i_* represent the slope and intercept of the carrier frequency expression in different periods, respectively.

### 4.2. Determine the Value of Δf_sin_

The selection principle of Δ*f*_sin_ is to fully realize the spread spectrum effect of “primary frequency modulation” and reduce the problem of “spectrum overlap” as much as possible. When *v*_m_(*t*) is a sinusoidal signal, and the value of Δ*f*_sin_ is 1, 2 and 3 kHz, the other parameters of the simulation are set as: *f*_s0_ = 10 kHz, *m* = 0.9, *f*_m_ = 30, *f*_1_ = 50 Hz. The distribution and characteristic analysis of the EMI peak in the simulation are shown in Table 4.

Based on the distribution characteristics of the conducted EMI spectrum under different conditions, Δ*f*_sin_ = 2 kHz is finally selected as the basis of the “secondary FM”.

### 4.3. Determine the Value Range of f_s1_ and f_s2_

According to Figure 5a, FB① and FB③ of the uneven part account for about 20% of the full frequency band, respectively, and the proportion of each component in FB② is less than 5% at most. Therefore, from the perspective of carrier frequency dispersion, the proportion of each component in the dispersed FB① and FB③ (within the length of 0.1Δ*f*_sin_ band) should not exceed 5%, so as to ensure that the EMI peak value of the dispersed frequency band is not higher than that of other frequency bands. The width of the frequency band before dispersion is 0.2Δ*f*_sin_, then the width of the frequency band after dispersion should be satisfied:

(20) $$\left\{\begin{array}{l} f_{s0}-0.8\Delta f_{\sin}-f_{s1}\geq 0.4\Delta f_{\sin}\\ f_{s2}-f_{s0}-0.8\Delta f_{\sin}\geq 0.4\Delta f_{\sin} \end{array}\right.$$

i.e., *f*_s1_ ≤ *f*_s0_ − 1.2Δ*f*_sin_; *f*_s2_ ≥ *f*_s0_ + 1.2Δ*f*_sin_

**Figure 9:**
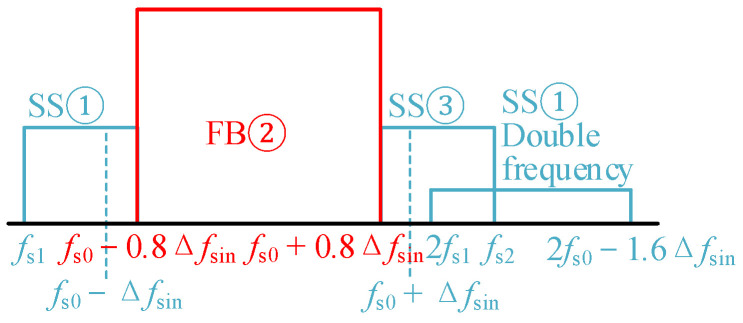
Schematic diagram of different frequency bands.

The lower and upper limits of the fundamental carrier frequency change are defined as *f*_smin_ and *f*_smax_, respectively. In order to reduce the overlap between “SS① double frequency” and “primary frequency modulation” original frequency band [*f*_s0_ − Δ*f*_sin_, *f*_s0_ + Δ*f*_sin_], as shown in Figure 9, 2 *f*_s1_ ≥ *f*_s0_ + Δ*f*_sin_ should be satisfied. For *f*_smax_, it is determined according to |*f*_smin_ − *f*_s0_| = |*f*_smax_ − *f*_s0_|, i.e.,:

(21) $$\left\{\begin{array}{l} f_{s\min}=(f_{s0}+\Delta f_{\sin})/2,\\ \left|f_{s\min}-f_{s0}|=|f_{s\max}-f_{s0}\right|\\ f_{s1}\geq f_{s\min},f_{s2}\leq f_{s\max} \end{array}\right.$$

Under the condition of Δ*f*_sin_ = 2 kHz, according to Equations (20) and (21), the variation range of *f*_s1_ is determined to be [6 kHz,7.6 kHz], and the variation range of *f*_s2_ is determined to be [12.4 kHz,14 kHz].

## 5. Experimental Results and Analysis

In this section, the “secondary FM” strategy and single periodic signal spread spectrum modulation are experimentally studied, and the effect of the traditional spread spectrum modulation strategy and the “secondary FM” on reducing the conducted EMI is analyzed. The input voltage ripple, output current and controller loss under different modulation strategies or different modulation parameters are measured.

**Figure 10:**
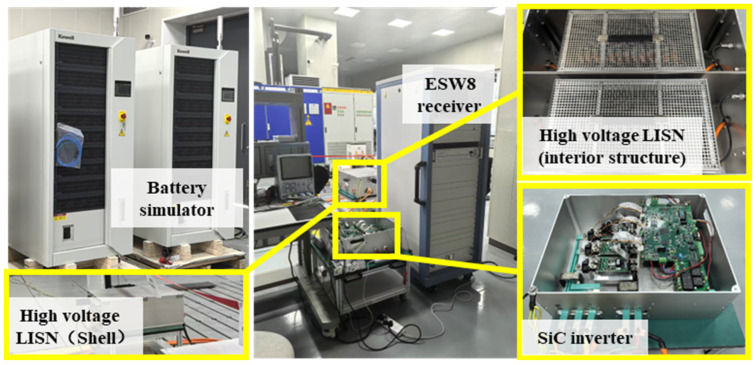
Schematic diagram of the experimental platform.

The experimental parameters are shown in Table 5, and the experimental platform is shown in Figure 10. The chip model of the control system is DSP28379, the model of the power semiconductor module is BSM600D12P3G001 SiC module, the DC side is powered by the battery simulator as the DC power supply and connected to the inverter through the NNHV 8123-800 high-voltage LISN of Rhode&Schwartz (Munich, Germany). The LISN load end is connected to the ESW8 receiver for testing the conducted EMI spectrum. The voltage and current ripple are measured by Yokogawa oscilloscope. The loss test is divided into two parts: the double-pulse test for measuring the characteristics of the inverter switch tube, and the loss test for measuring the efficiency of the inverter. The double-pulse test is completed by the Edison DPT2K04B (Dongguan, China) power device dynamic parameter measurement instrument, and the loss test is completed by the Yokogawa (Tokyo, Japan) WT5000 power analyzer.

### 5.1. Experimental Parameter Design of “Secondary FM” Modulation Strategy

**Figure 11:**
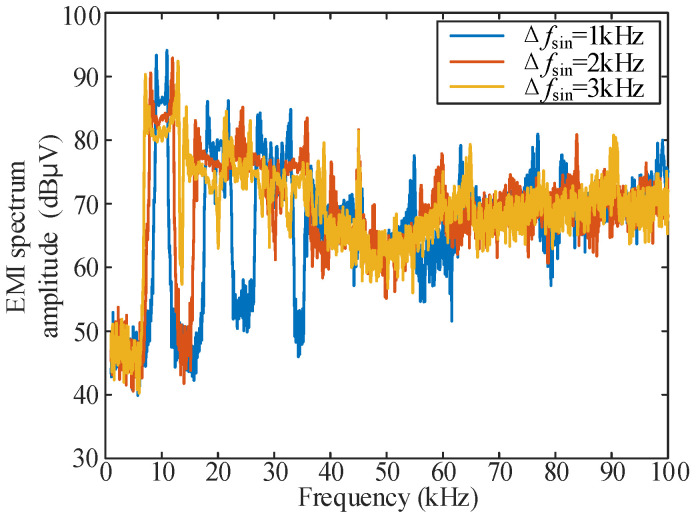
Corresponding EMI spectrum of *v*_m_(*t*)-sine with different Δ*f*_sin_.

The EMI spectrum distribution characteristics of a single sinusoidal signal with spread spectrum modulation in three cases of Δ*f*_sin_ = 1, 2 and 3 kHz are tested experimentally, and the results are shown in Figure 11.

According to Figure 11, for Δ*f*_sin_ = 1, 2, 3 kHz, the lowest amplitude of the EMI spectrum of the spread spectrum modulation of the sine signal in [*f*_s0_ − Δ*f*_sin_, *f*_s0_ + Δ*f*_sin_] band is shown in Table 6. The spectrum distribution characteristics under the three conditions in Figure 11 are highly similar to the analysis in Table 4, and according to the results shown in Figure 11 and Table 6, it can be seen that the selection of Δ*f*_sin_ = 2 kHz as the basic condition of “secondary FM” is still reasonable in the actual experiment.

### 5.2. Comparison of EMI Experimental Results

In this section, the traditional period spread spectrum modulation strategy and the “secondary FM” strategy proposed in this paper are tested, to verify and compare the effects of different strategies on reducing the conducted EMI peak.

#### 5.2.1. EMI Results of Spread Spectrum Modulation of Single Periodic Signal

**Figure 12:**
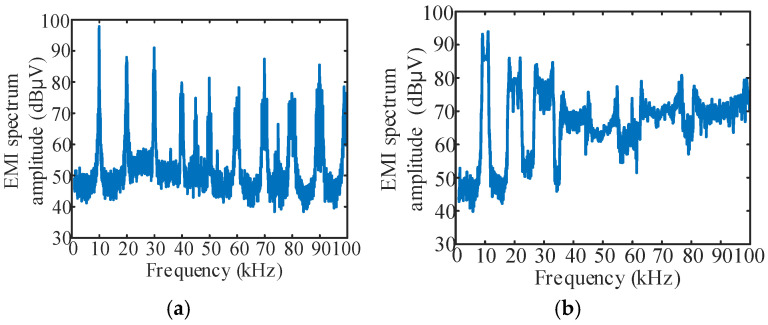

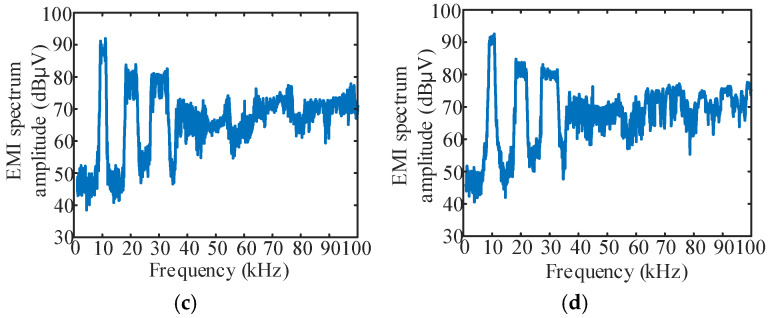
EMI spectrum of fixed carrier frequency modulation and spread spectrum modulation at Δ*f* = 1 kHz: (**a**) The carrier frequency is fixed at 10 kHz; (**b**) *v*_m_(*t*)-sine, Δ*f* = 1 kHz; (**c**) *v*_m_(*t*)-triangle, Δ*f* = 1 kHz; (**d**) *v*_m_(*t*)-sawtooth, Δ*f* = 1 kHz.

In order to verify the effect of spread spectrum modulation of a single periodic signal on reducing the conducted EMI spike, the EMI spectrum of fixed carrier frequency modulation and *v*_m_(*t*)-sine, *v*_m_(*t*)-triangle, *v*_m_(*t*)-sawtooth spread spectrum modulation at Δ*f* = 1 kHz was tested, and the results are shown in Figure 12.

It can be seen from the figure that the peak value of conducted EMI under a fixed carrier frequency of 10 kHz is 98.03 dBμV, and the peak value of conducted EMI corresponding to sine, triangle and sawtooth signals under Δ*f* = 1 kHz is 94.11 dBμV, 92.1 dBμV and 92.52 dBμV, respectively, which are lower than those under a fixed carrier frequency modulation.

**Figure 13:**
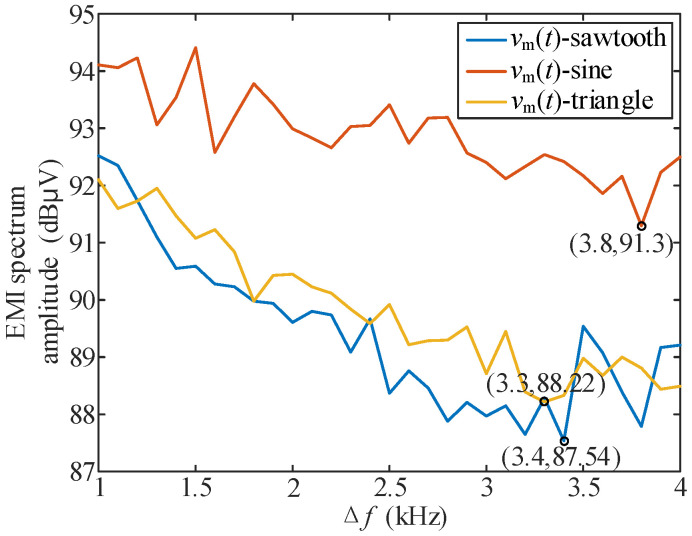
EMI spectrum peaks corresponding to different types of periodic signals at different Δ*f.*

In order to analyze the influence of *v*_m_(*t*) and Δ*f* on conducted EMI, *v*_m_(*t*)-sine, *v*_m_(*t*)-triangle, *v*_m_(*t*)-sawtooth spread spectrum modulation were tested, respectively, and the conducted EMI peak values corresponding to different conditions of Δ*f* = 1 kHz:0.1 kHz:4 kHz (that is, all cases of carrier frequency fluctuation in the range of 6 kHz to 14 kHz) were measured. A total of 93 groups of data were collected. The measurement results are shown in Figure 13, where the lowest peak value of conducted EMI and the corresponding Δ*f* under spread spectrum modulation are also marked.

Figure 13 shows that the EMI peak does not decrease monotonically with the increase of Δ*f*, corresponding to the “spectrum overlap” phenomenon analyzed; therefore, the purpose of reducing the conducted EMI peak cannot be achieved by simply increasing Δ*f*.

#### 5.2.2. Analysis of EMI Experimental Results of “Secondary FM” Spread Spectrum Modulation Strategy

**Figure 14:**
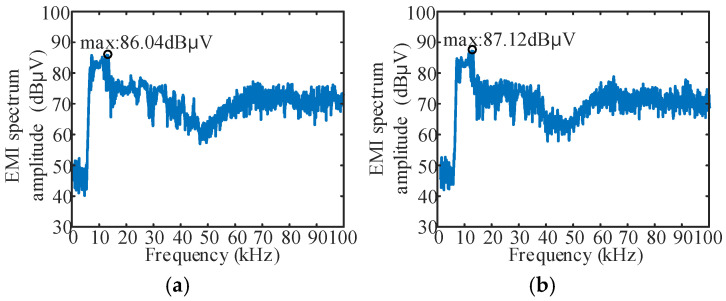
“Secondary FM” results: (**a**) *f*_s1_ = 6.5 kHz, *f*_s2_ = 13.9 kHz; (**b**) *f*_s1_ = 6.7 kHz, *f*_s2_ = 13.3 kHz.

At Δ*f*_sin_ = 2 kHz, the FB① and FB③ were dispersed as described in Section 4. That is, the frequency band of the sinusoidal signal is [8.4 kHz, 11.6 kHz], the [8 kHz, 8.4 kHz] frequency band is separately dispersed into [*f*_s1_, 8.4 kHz], and the [11.6 kHz, 12 kHz] frequency band is dispersed into the [11.6 kHz, *f*_s2_] frequency band. The results under different combinations of *f*_s1_ and *f*_s2_ parameters were tested, and partial results are shown in Figure 14.

By comparing the results of Figure 13 and Figure 14a, the “secondary FM” strategy can achieve a better EMI peak reduction effect than the single periodic signal spread spectrum modulation in the *f*_s_ range of 6 k~14 kHz. The optimal effect of the “secondary FM” method is reduced by an additional 1.5 dBμV on the basis of 87.54 dBμV in Figure 13. The comparison of results in Figure 13 and Figure 14b shows that with the same or lower Δ*f*, “secondary FM” can also achieve better EMI peak reduction effect.

### 5.3. Input Voltage and Output Current Ripple

**Figure 15:**
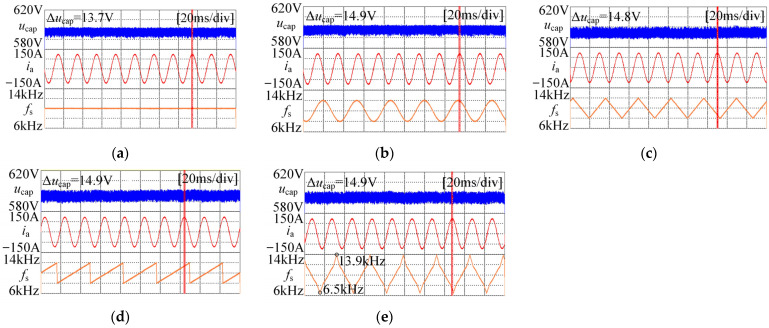
Input DC voltage, output current and corresponding carrier frequency variation waveforms under different conditions: (**a**) The carrier frequency is fixed at 10 kHz; (**b**) *v*_m_(*t*)-sine, Δ*f* = 2 kHz; (**c**) *v*_m_(*t*)-triangle, Δ*f* = 2 kHz; (**d**) *v*_m_(*t*)-sawtooth, Δ*f* = 2 kHz; (**e**) *f*_s1_ = 6.5 kHz, *f*_s2_ = 13.9 kHz, secondary FM.

In this section, the input voltage ripple and output current of different modulation strategies are tested, respectively, and the results are shown in Figure 15. It can be seen from the figure that for input voltage ripple, the maximum difference of voltage ripple under different conditions is only 1.2 V, which is not much different from the input voltage ripple under fixed carrier frequency. Compared with 600 V DC bus voltage, the increase of ripple is only 0.2%.

As for the output current, it can be seen from Figure 15 that there is no obvious distortion of the output phase current, indicating that the spread spectrum modulation strategy will not significantly affect the quality of the output current.

### 5.4. Loss Testing

**Figure 16:**
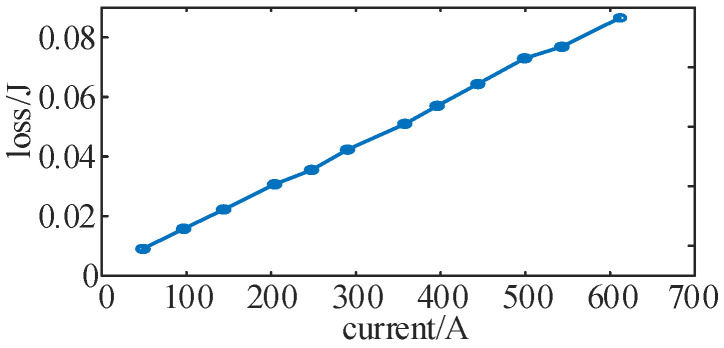
Changing trend of switching loss with current.

In this section, the double-pulse experiment is carried out to test the switching tube loss data under 12 different switching currents, starting from 50 A and increasing by 50 A until 600 A each time. The relationship between the corresponding current and switching loss is shown in Figure 16.

According to the results shown in Figure 16, each parameter in Equation (19) is identified and obtained:

(22) $$E_{\mathrm{sw}}=1\times 10^{-9}{\left[i(t)\right]}^{2}+1\times 10^{-4}[i(t)]+0.0022$$

Under the experimental parameters shown in Table 5, combined with Equations (3) and (22), the expression of switching loss in a single carrier cycle at different times and different currents can be obtained as:

(23) $$E_{\mathrm{sw}}=1\times 10^{-9}(i_{a}^{2}+i_{b}^{2}+i_{c}^{2})+1\times 10^{-4}(|i_{a}|+|i_{b}|+|i_{c}|)+0.0066$$

The total switching loss of the inverter per unit time can be obtained by accumulating the switching loss at different times. Taking the Δ*f* = 1 kHz condition as an example, the numerical theoretical evaluation of switching loss within 1 s for different modulation strategies is shown in Table 7.

**Figure 17:**
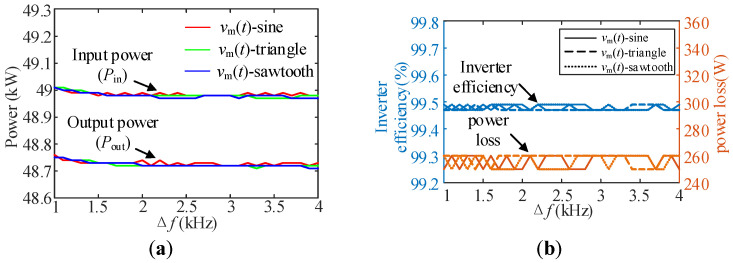
Inverter input and output power, efficiency and loss under different conditions: (**a**) The corresponding inverter input and output power of different periodic signals at different Δ*f*; (**b**) Inverter efficiency and power loss corresponding to different period signals at different Δ*f.*

When *v*_m_(*t*) is three kinds of periodic signals, the input and output power, efficiency and loss under different Δ*f* are measured by the power analyzer, and the results are shown in Figure 17. The figure contains the experimental data of three kinds of periodic signals under the condition of Δ*f* = 1 kHz:0.1 kHz:4 kHz.

**Figure 18:**
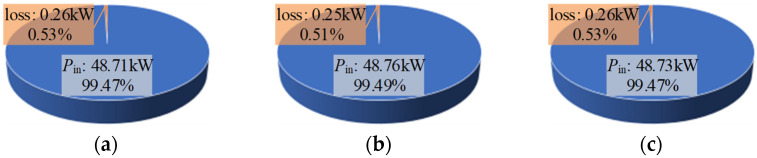
Loss results under three typical modulation strategies: (**a**) Fixed carrier frequency modulation strategy, *f*_s_ = 10 kHz; (**b**) *v*_m_(*t*)-sine, Δ*f* = 1 kHz; (**c**) Secondary FM, *f*_s1_ = 6.5 kHz, *f*_s2_ = 13.9 kHz.

In addition, the loss test results of three typical modulation strategies are selected for presentation, as shown in Figure 18. Figure 17 and Figure 18 show that there is almost no difference between different types of spread spectrum modulation strategies and fixed carrier frequency modulation in terms of loss and efficiency. In addition, the loss results tested by the power analyzer are very close to the loss evaluation results fitted based on the two-pulse experiment in Table 7, which verifies the effectiveness of the method of indirectly evaluating switching loss through the number of carriers included in the unit time in the spread spectrum modulation strategy. Therefore, in general, when designing the spread spectrum modulation strategy, its impact on the loss can be ignored under the premise that the average carrier frequency does not change much.

## 6. Conclusions

In this paper, the factors affecting the conducted EMI of the motor controller are analyzed, and the impact level of the spread spectrum modulation strategy on the input DC voltage ripple, output AC current ripple, and controller loss is theoretically evaluated. On this basis, a “secondary FM” spread spectrum modulation strategy is further proposed. Research shows that:

(1) In the single periodic signal spread spectrum modulation, the sawtooth signal has the best suppression effect on EMI peak;

(2) When *f*_s0_ is fixed, the influence of spread spectrum modulation on input voltage ripple and stator flux fluctuation mainly depends on Δ*f*. The larger Δ*f* is, the larger input voltage ripple and stator flux fluctuation are, while the type of periodic signal and *f*_m_ have little effect on them.

(3) The influence of spread spectrum modulation strategy on controller loss is negligible.

(4) In the limited range of carrier frequency variation, compared with the traditional period spread spectrum modulation strategy, the “secondary FM” strategy can further reduce the conducted EMI of the motor controller, the reduced amplitude of conducted EMI is 1.5 dBμV, and this method does not have significant impact on the ripple, loss and other indicators.

Compared with the existing research, the influence of spread spectrum modulation strategy on the input/output performance and loss of the inverter is theoretically analyzed in this paper. The existing research on these indicators under the spread spectrum modulation strategy is usually directly through the experiment, which lacks theoretical analysis. This paper theoretically makes up for this deficiency.

## Figures and Tables

**Table 1:** Variations of voltage ripple and flux fluctuation when different parameters increase.

Increased Parameters	*m*	*f*_1_	*f*_s0_	*f*_m_	Δ*f*
*u*_cap_r_	Increase	Decrease	Decrease	—	Increase
*ψ*_rip_rms_	Increase	Unrelated	Decrease	—	Slightly increase

**Table 2:** Number of carriers within 1 s for different modulation strategies.

Modulation Strategy	Number of Carriers
The carrier frequency is fixed at 10 kHz	10,000
*v*_m_(*t*)-sine, Δ*f* = 1 kHz:0.1 kHz:4 kHz	10,048~10,056
*v*_m_(*t*)-triangle, Δ*f* = 1 kHz:0.1 kHz:4 kHz	10,050~10,053
*v*_m_(*t*)-sawtooth, Δ*f* = 1 kHz:0.1 kHz:4 kHz	10,050

**Table 3:** *v*_EMI_ spectrum peak of each band under different conditions (unit: dB).

Type of *v*_m_(*t*)	FB①	FB②	FB③
Sine	59.10	55.49	61.26
Triangle	56.10	58.75	58.28
Sawtooth	55.00	59.10	57.96

**Table 4:** Distribution of EMI spikes in each frequency band under different Δ*f*_sin_ when *v*_m_(*t*) is a sinusoidal signal.

Δ*f*_sin_	FB①	FB②	FB③	Analysis of Spectral Distribution Characteristics
1 kHz	62.81	63.52	62.94	The EMI spectrum dispersion is low, the energy is still relatively concentrated, and some spikes will appear in the FB②.
2 kHz	60.13	57.72	61.04	The dispersion degree of the EMI spectrum increases, the spike is further weakened, and the EMI peak in FB② decreases significantly.
3 kHz	59.10	55.49	61.26	The EMI spectrum is further dispersed, but the weakening degree of the peak of FB② is limited, and the 2(*f*_s0_ − Δ*f*_sin_) is close to *f*_s0_ + Δ*f*_sin_. The “secondary FM” on this basis easily causes “spectrum overlap”.

**Table 5:** Experimental parameter settings.

Parameter	Comment	Value
*U*_dc_	DC Bus Voltage	600 V
*m*	Modulation Index	0.9
*Z*_L_	Load Impedance	2.75 Ω + 1 mH
*f*_1_	Output Fundamental Frequency	50 Hz
*f*_m_	Periodic Signal *v*_m_(*t*) Frequency	30 Hz
*f*_s0_	Center Carrier Frequency	10 kHz

**Table 6:** For Δ*f*_sin_ = 1, 2, 3 kHz, the lowest EMI amplitude of the spread spectrum modulation of the sine signal in [*f*_s0_ − Δ*f*_sin_, *f*_s0_ + Δ*f*_sin_].

Δ*f*_sin_	[*f*_s0_ − Δ*f*_sin_, *f*_s0_ + Δ*f*_sin_]	EMI Minimum Amplitude
1 kHz	[9 kHz, 11 kHz]	85.4 dBμV
2 kHz	[8 kHz, 12 kHz]	81.9 dBμV
3 kHz	[7 kHz, 13 kHz]	79.7 dBμV

**Table 7:** Number of carriers and switching losses within 1 s for different modulation strategies.

Modulation Strategy	Number of Carriers	E_loss_/W
*f*_s_ = 10 kHz, fixed carrier frequency	10,000	281.31
*v*_m_(*t*)-sine, Δ*f* = 1 kHz	10,050	282.72
*v*_m_(*t*)-triangle, Δ*f* = 1 kHz	10,051	282.74
*v*_m_(*t*)-sawtooth, Δ*f* = 1 kHz	10,050	282.69

## Data Availability

Data is contained within the article.

## Abbreviations

**Variable**	**Comment**	**Variable**	**Comment**
*U*_dc_	DC bus voltage	*L*_dc_	Stray inductance
*i*_L_	Current of stray inductor branch	***U*_ref_**	Reference voltage vector
*i*_Cap_	Capacitance current	*u*_Cap_	DC side input voltage
*i*_dc_	DC side input current	*C*_dc_	DC side support capacitance
*s*_a_, *s*_b_, *s*_c_	Switching state of the three-phase bridge arm	*ω*	Angular frequency of reference voltage
*i*_a_, *i*_b_, *i*_c_	Three-phase load current	*m*	Modulation index
*u*_Cap_r_	DC side voltage ripple	*R*, *L*	Load resistance and inductance
*Z*_L_	Load impedance	*f*_1_	Output fundamental frequency
*φ*_0_	Initial phase of the phase A voltage	*I*_o_	Amplitude of output phase current
*φ*_L_	Power factor angle of load	*f*_m_	Frequency of periodic signal
*u*_d_, *u*_q_	Voltage component of d and q-axis	*ψ*_rip_rms_	Effective value of flux fluctuation
*f*_s_	Carrier frequency	*f*_s0_	Centre carrier frequency
*v*_m_(*t*)	Periodic signal	*T*_s_	Carrier cycle
Δ*f*_s_	Maximum offset frequency of single signal periodic spread spectrum modulation strategy	*θ*	The angle between the voltage reference vector ***U*_ref_** and the α axis
Δ*f*_sin_	“Primary frequency modulation” spread width	*ψ*_rd_, *ψ*_rq_	Flux fluctuation component of d and q-axis
***U*_0_**~***U*_7_**	SVPWM basic vector	*T*_m_	Signal period

## References

[1] Qiu Z., Chen Y., Cheng H.Q., Liu X., Gu F. (2022). Periodic Harmonic Spread Spectrum Modulation for High-Frequency Sideband Vibro-Acoustic Suppression in Permanent Magnet Synchronous Motor. Trans. China Electrotech. Soc..

[2] Huang J., Li K. (2022). Suppressing the Maximum EMI Spectral Peak Through Asynchronous Carriers in the Three-Phase Inverter with the Periodic CFM. IEEE Trans. Power Electron..

[3] Chen J., Jiang D., Sun W., Shen Z., Zhang Y. (2020). A Family of Spread-Spectrum Modulation Schemes Based on Distribution Characteristics to Reduce Conducted EMI for Power Electronics Converters. IEEE Trans. Ind. Appl..

[4] Huang Y., Xu Y., Zhang W., Zou J. (2019). Hybrid RPWM Technique Based on Modified SVPWM to Reduce the PWM Acoustic Noise. IEEE Trans. Power Electron..

[5] Qi C., Chen X., Mou X. (2012). A Hybrid Spread Spectrum Modulation Technique for PWM Inverters. Proc. CSEE.

[6] Gamoudi R., Chariag D.E., Sbita L. (2018). A Review of Spread-Spectrum-Based PWM Techniques—A Novel Fast Digital Implementation. IEEE Trans. Power Electron..

[7] Mina K., Hwa-Pyeong P., Jee-Hoon J. (2022). Spread Spectrum Technique with Random-Linear Modulation for EMI Mitigation and Audible Noise Elimination in IH Appliances. IEEE Trans. Ind. Electron..

[8] Xu Y., Yuan Q., Zou J., Li Y. (2012). Analysis of Triangular Periodic Carrier Frequency Modulation on Reducing Electromagnetic Noise of Permanent Magnet Synchronous Motor. IEEE Trans. Magn..

[9] Shan Y., Pei X., Sun T., Zhang M., Zhou P., Jiang D. (2022). Space Spread-Spectrum Strategy for MMC to Reduce the Conducted EMI. IEEE Trans. Ind. Electron..

[10] Lee K., Shen G., Yao W., Lu Z. (2017). Performance Characterization of Random Pulse Width Modulation Algorithms in Industrial and Commercial Adjustable-Speed Drives. IEEE Trans. Ind. Appl..

[11] Kumar A.C.B., Narayanan G. (2016). Variable-Switching Frequency PWM Technique for Induction Motor Drive to Spread Acoustic Noise Spectrum with Reduced Current Ripple. IEEE Trans. Ind. Appl..

[12] Szwarc K.J., Szczepankowski P., Nieznanski J., Swinarski C., Usoltsev A., Strzelecki R. (2020). Hybrid Modulation for Modular Voltage Source Inverters with Coupled Reactors. Energies.

[13] Kapil P.N., Sant A.V. Comparison of Various Modulation Techniques for Conventional, Quasi and Switched Inductor Z-Source Inverter Topologies. Proceedings of the 2023 IEEE 2nd Industrial Electronics Society Annual On-Line Conference (ONCON).

[14] Stepenko S., Husev O., Vinnikov D., Roncero-Clemente C., Pimentel S.P., Santasheva E. (2019). Experimental Comparison of Two-Level Full-SiC and Three-Level Si-SiC Quasi-Z-Source Inverters for PV Applications. Energies.

[15] Stepenko S., Husev O., Vinnikov D., Fesenko A., Matiushkin O. (2020). Feasibility Study of Interleaving Approach for Quasi-Z-Source Inverter. Electronics.

[16] Fuentes C.D., Müller M., Bernet S., Kouro S. (2021). SiC-MOSFET or Si-IGBT: Comparison of Design and Key Characteristics of a 690 V Grid-Tied Industrial Two-Level Voltage Source Converter. Energies.

[17] Pei X., Chen C., Kang Y. (2014). Analysis of Voltage Ripple and Design for DC-Link Capacitor in Three-Phase Voltage Source Inverters. Trans. China Electrotech. Soc..

[18] Casadei D., Serra G., Tani A., Zarri L. (2004). Theoretical and experimental analysis for the RMS current ripple minimization in induction motor drives controlled by SVM technique. IEEE Trans. Ind. Electron..

[19] Casadei D., Serra G., Tani A., Zarri L. (2009). Optimal Use of Zero Vectors for Minimizing the Output Current Distortion in Matrix Converters. IEEE Trans. Ind. Electron..

[20] Chen J., Jiang D., Shen Z., Sun W., Fang Z. (2020). Uniform Distribution Pulsewidth Modulation Strategy for Three-Phase Converters to Reduce Conducted EMI and Switching Loss. IEEE Trans. Ind. Electron..

[21] Johnson S., Zane R. (2005). Custom Spectral Shaping for EMI Reduction in High-Frequency Inverters and Ballasts. IEEE Trans. Power Electron..

[22] Li J., Dang E., Fan Y., Dong H., Liu J. (2024). A Hybrid Three-Level Active-Neutral-Point-Clamped Zero-Voltage Transition Soft-Switching Converter with Silicon Carbide and Silicon Devices. Trans. China Electrotech. Soc..

